# Mitochondrial retrograde signaling in gastric cancer: mtDNA mutations, ROS-driven NF-κB/HIF-1α activation, and Warburg effect amplification

**DOI:** 10.3389/fphys.2026.1931732

**Published:** 2026-09-08

**Authors:** Yanyan Bai, Wenbin Chen

**Affiliations:** Shandong Provincial Hospital Affiliated to Shandong First Medical University, Jinan, China

**Keywords:** gastric cancer, metabolic reprogramming, mitochondrial retrograde signaling, mtDNA mutations, reactive oxygen species

## Abstract

Mitochondrial retrograde signaling serves as a critical communication axis that links mitochondrial dysfunction to nuclear gene expression, shaping key pathways in gastric carcinogenesis. Gastric tumors frequently exhibit somatic mtDNA mutations, impaired oxidative phosphorylation, and elevated reactive oxygen species (ROS), collectively driving transcriptional reprogramming through activation of NF-κB and stabilization of HIF-1α. These retrograde signals promote inflammation, metabolic reprogramming, and resistance to apoptosis, ultimately reinforcing the Warburg phenotype characterized by enhanced aerobic glycolysis and lactate production. This review synthesizes current evidence on how mtDNA mutations, ROS-dependent transcription factor activation, and mitochondrial–nuclear metabolic crosstalk converge to promote malignant transformation. By integrating insights from mitochondrial biology, cancer genetics, and metabolic regulation, we outline a mechanistic framework that highlights mitochondrial retrograde signaling as a promising therapeutic target in gastric cancer.

## 1 Introduction

Gastric carcinogenesis typically follows the classical Correa cascade, beginning with H. pylori–induced chronic gastritis and progressing through atrophic gastritis, intestinal metaplasia, dysplasia, and ultimately invasive carcinoma (Figueiredo et al., 2017; Joshi and Badgwell, 2021). Despite advances in diagnostic and therapeutic strategies, prognosis for advanced gastric cancer remains poor, with five-year survival rates below 30% for metastatic disease (Zeng and Jin, 2022). The marked molecular heterogeneity of gastric cancer, encompassing diverse histological and genomic subtypes, continues to challenge the development of effective targeted therapies (Chia and Tan, 2016; Machlowska et al., 2020). Understanding the core molecular mechanisms that drive gastric carcinogenesis is therefore essential for identifying new therapeutic opportunities.

Mitochondria, traditionally viewed as cellular powerhouses generating ATP through oxidative phosphorylation, are now recognized as central regulators of apoptosis, calcium homeostasis, reactive oxygen species (ROS) signaling, and metabolic reprogramming (Wallace, 2012; Weinberg and Chandel, 2015). Beyond energy production, mitochondria act as signaling organelles that communicate their functional status to the nucleus through retrograde signaling pathways (Ryan and Hoogenraad, 2007; Melber and Haynes, 2018), enabling adaptive transcriptional responses under conditions of mitochondrial stress.The concept of mitochondrial dysfunction in cancer has evolved substantially since Warburg’s early observations of aerobic glycolysis in tumor cells (Warburg, 1956; Vander Heiden et al., 2009). Rather than reflecting simple respiratory defects, mitochondrial dysfunction in cancer involves coordinated alterations in mitochondrial dynamics, metabolism, and signaling that actively promote oncogenesis (Boland et al., 2013; Desideri et al., 2015). Dysfunctional mitochondria generate retrograde signals that reshape nuclear gene expression toward a pro-tumorigenic phenotype. Mitochondrial DNA (mtDNA), a 16.6-kb genome encoding essential OXPHOS components, exhibits a mutation rate 10- to 17-fold higher than nuclear DNA due to limited repair capacity and proximity to ROS generated during respiration (Wallace, 2012; Copeland and Longley, 2014; Darfarin and Pluth, 2025). Somatic mtDNA mutations are frequent across cancers (Brandon et al., 2006; Ju et al., 2014), including gastric tumors where they disrupt electron transport chain function, elevate ROS production, and activate oncogenic retrograde signaling pathways (Lee et al., 2014; Tanprasert et al., 2022).

ROS, produced mainly at complexes I and III of the electron transport chain, serve as signaling molecules at physiological levels (Sullivan and Chandel, 2014; Reczek and Chandel, 2017). Excessive ROS generated during mitochondrial dysfunction induces oxidative stress and activates redox-sensitive transcription factors, most notably NF-κB and HIF-1α (Morgan and Liu, 2011; Korbecki et al., 2021). NF-κB regulates inflammation, immunity, and cell survival, and its activation by mitochondrial ROS links mitochondrial stress to pro-inflammatory and pro-survival transcriptional programs. In gastric cancer, H. pylori-induced inflammation synergizes with mitochondrial ROS to sustain NF-κB activation and promote tumor progression (Rhee, 2000; Duan et al., 2025).

HIF-1α, the master regulator of hypoxic responses, is stabilized under normoxia by mitochondrial ROS through inhibition of prolyl hydroxylases. This stabilization enables cancer cells to activate hypoxia-like transcriptional programs, enhancing glycolysis, angiogenesis, and metastatic potential. Crosstalk between HIF-1α and NF-κB further amplifies these oncogenic responses (Rius et al., 2008; Korbecki et al., 2021).

The Warburg effect enhanced glycolysis and lactate production despite oxygen availability represents a metabolic hallmark of cancer that supports biosynthesis and confers resistance to apoptosis. In gastric cancer, this phenotype is actively reinforced by mitochondrial retrograde signaling through ROS-dependent activation of HIF-1α and related transcription factors (Wen et al., 2019; Jiang and Ye, 2025). Beyond histological diversity, gastric cancer also exhibits distinct molecular subtypes such as EBV-positive, MSI-high, genomically stable, and chromosomal instability tumors each associated with characteristic metabolic behaviors. EBV-positive and CIN tumors frequently display enhanced glycolysis and elevated ROS production, whereas MSI-high tumors show more variable metabolic activity with reduced reliance on OXPHOS (Adam et al., 2021). Genomically stable tumors, often enriched for RHOA mutations and EMT features, demonstrate pronounced mitochondrial stress and metabolic reprogramming. These subtype-specific metabolic patterns provide a biological foundation for understanding how mitochondrial dysfunction and retrograde signaling contribute to gastric carcinogenesis (Ning et al., 2025).

This review synthesizes current knowledge regarding mitochondrial retrograde signaling in gastric cancer, with particular emphasis on three interconnected mechanisms: (1) somatic mtDNA mutations as initiators of retrograde signaling; (2) ROS-driven activation of NF-κB and HIF-1α and the resulting nuclear reprogramming; and (3) amplification of the Warburg phenotype through mitochondrial-nuclear communication. By integrating insights from molecular oncology, mitochondrial biology, and cancer metabolism, we present a comprehensive framework for understanding how mitochondrial dysfunction drives gastric carcinogenesis and identify potential therapeutic targets for precision medicine approaches.

## 2 Mitochondrial structure and function in normal physiology

Mitochondria are double-membrane organelles present in virtually all eukaryotic cells, originating from an ancient endosymbiotic event between an α-proteobacterium and an archaeal host (Lane and Martin, 2010; Gray, 2012). This evolutionary origin explains the retention of a separate mitochondrial genome and the semi-autonomous nature of mitochondrial function. The outer mitochondrial membrane, containing porins that allow passage of molecules up to approximately 5 kDa, encloses the inner mitochondrial membrane, which is highly impermeable and contains the protein complexes responsible for oxidative phosphorylation.

The inner mitochondrial membrane is organized into cristae, invaginations that dramatically increase surface area for electron transport chain complexes (Mannella, 2006; Cogliati et al., 2013). The matrix, enclosed by the inner membrane, contains the mitochondrial genome, ribosomes, and enzymes essential for the tricarboxylic acid (TCA) cycle, fatty acid oxidation, and amino acid metabolism (Nunnari and Suomalainen, 2012). This compartmentalization creates specialized microenvironments that optimize metabolic efficiency while enabling precise regulation of mitochondrial function.

The electron transport chain, comprising complexes I-V embedded in the inner mitochondrial membrane, generates the proton gradient that drives ATP synthesis through oxidative phosphorylation. Complexes I (NADH dehydrogenase) and II (succinate dehydrogenase) oxidize NADH and FADH2, respectively, transferring electrons to ubiquinone. Complex III (cytochrome bc1 complex) and complex IV (cytochrome c oxidase) subsequently transfer electrons to molecular oxygen, the final electron acceptor, while pumping protons from the matrix to the intermembrane space (Korzeniewski and Froncisz, 1991; Brand, 2005).

This chemiosmotic coupling, as originally proposed by Peter Mitchell, generates an electrochemical gradient across the inner mitochondrial membrane that drives ATP synthesis by complex V (ATP synthase) (Mitchell, 1966). Under normal physiological conditions, oxidative phosphorylation generates approximately 90% of cellular ATP, with the remainder produced through glycolysis in the cytosol. The efficiency of this process enables aerobic organisms to extract significantly more energy from glucose (approximately 36–38 ATP per glucose) compared to anaerobic glycolysis (2 ATP per glucose).

Mitochondria are highly dynamic organelles that undergo continuous cycles of fusion and fission, processes that maintain mitochondrial quality and adapt cellular metabolism to changing energetic demands (Westermann, 2010; Chan, 2012). Mitochondrial fusion, mediated by mitofusins 1 and 2 (MFN1/2) and optic atrophy 1 (OPA1), enables content mixing and complementation between mitochondria. Mitochondrial fission, regulated by dynamin-related protein 1 (DRP1) and its receptors, facilitates mitochondrial segregation and quality control through mitophagy (Chen et al., 2003; Losón et al., 2013). The balance between fusion and fission determines mitochondrial morphology, with elongated networks associated with efficient OXPHOS and fragmented mitochondria characteristic of metabolic stress or dysfunction (Gomes et al., 2011). Mitophagy is a central component of mitochondrial quality control, selectively removing damaged or depolarized mitochondria to preserve cellular homeostasis. In the canonical PINK1–Parkin pathway, loss of mitochondrial membrane potential prevents PINK1 import and degradation, resulting in its stabilization on the outer mitochondrial membrane. PINK1 phosphorylates ubiquitin and the E3 ubiquitin ligase Parkin, thereby activating Parkin and promoting the ubiquitination of outer-mitochondrial-membrane proteins. These ubiquitin signals recruit autophagy receptors and facilitate autophagosome formation, followed by lysosomal degradation of the damaged mitochondrion (Bingol and Sheng, 2016). In parallel, receptor-mediated mitophagy pathways involving BNIP3, NIX, and FUNDC1 operate under hypoxia or metabolic stress. Through these mechanisms, mitophagy limits excessive ROS production, prevents release of pro-apoptotic factors, and preserves mitochondrial network integrity, functioning in concert with fusion–fission dynamics to regulate mitochondrial quality (Yang et al., 2025).

Mitochondrial retrograde signaling refers to the communication of mitochondrial status to the nucleus, resulting in adaptive changes in nuclear gene expression (Butow and Avadhani, 2004). Unlike anterograde signaling, wherein nuclear-encoded proteins are imported into mitochondria, retrograde signaling transmits information about mitochondrial functional state, stress, or damage to elicit appropriate cellular responses. This bidirectional communication ensures coordination between mitochondrial and nuclear genomes, which together encode the approximately 1,500 proteins comprising the mitochondrial proteome (Cotter et al., 2004; Calvo et al., 2016).

The prototypical retrograde response was initially characterized in yeast, where mitochondrial dysfunction activates the transcription factor RTG1, leading to upregulation of genes encoding TCA cycle enzymes and other metabolic proteins (Epstein et al., 2001). In mammalian cells, retrograde signaling is considerably more complex, involving multiple parallel pathways that respond to diverse mitochondrial perturbations including membrane depolarization, ROS generation, calcium release, mtDNA damage, and protein misfolding (Durieux et al., 2011).

Several key pathways mediate mitochondrial retrograde signaling in mammalian cells. The mitochondrial unfolded protein response (UPRmt) is activated by accumulation of misfolded proteins in the mitochondrial matrix, triggering activation of the transcription factor ATFS-1, which contains both a mitochondrial targeting sequence and a nuclear localization signal (Haynes et al., 2007; Nargund et al., 2015). Under conditions of mitochondrial import stress, ATFS-1 accumulates in the nucleus and activates transcription of mitochondrial chaperones and proteases to restore protein homeostasis.

Calcium represents another important retrograde signaling molecule, as mitochondria function as cellular calcium buffers that regulate cytosolic calcium levels (Rizzuto et al., 2012; Bravo‐Sagua et al., 2017). Mitochondrial calcium uptake through the mitochondrial calcium uniporter (MCU) activates mitochondrial dehydrogenases, stimulating OXPHOS to meet increased energy demands. Conversely, excessive mitochondrial calcium accumulation can trigger opening of the mitochondrial permeability transition pore (mPTP), releasing pro-apoptotic factors and calcium into the cytosol (Bernardi et al., 2015; Halestrap and Richardson, 2015).

ROS generated during mitochondrial respiration function as signaling molecules that modulate diverse cellular processes through reversible oxidation of redox-sensitive cysteine residues in target proteins. At physiological levels, mitochondrial ROS activate adaptive responses including antioxidant gene expression through Nrf2 activation and metabolic reprogramming through HIF-1α stabilization (Bell et al., 2007; Winterbourn, 2008). Excessive ROS production, however, leads to oxidative damage of lipids, proteins, and DNA, while simultaneously activating inflammatory and stress response pathways.

Importantly, mitochondrial retrograde signaling should be viewed as a mechanistically causal process rather than a passive correlate of mitochondrial dysfunction. Experimental systems such as cybrids, mtDNA-depleted ρ0 cells, and targeted disruption of membrane potential demonstrate that mitochondrial stressors including ROS accumulation, altered calcium flux, impaired OXPHOS, and UPRmt activation are sufficient to induce nuclear transcriptional programs involving NF-κB, HIF-1α, and the mammalian UPRmt transcription factors ATF4, ATF5, and CHOP (Keerthiga et al., 2021). These pathways drive metabolic reprogramming, antioxidant responses, and survival signaling even in the absence of additional oncogenic stimuli, indicating a link and an effect relationship. Furthermore, interventions that block retrograde signaling, including mitochondria-targeted antioxidants and calcium chelators, can reverse downstream features such as the Warburg phenotype, invasive behavior, and chemoresistance. These findings support the view that retrograde signaling actively contributes to gastric carcinogenesis rather than merely accompanying mitochondrial dysfunction (Yang and Kim, 2019).

Metabolites generated within mitochondria also serve as retrograde signaling molecules. Acetyl-CoA, produced through pyruvate dehydrogenase activity in the mitochondrial matrix, regulates protein acetylation after export to the cytosol and nucleus (Wellen et al., 2009; Shi and Tu, 2015). Similarly, succinate and fumarate, TCA cycle intermediates that accumulate upon disruption of OXPHOS, inhibit prolyl hydroxylases and thereby stabilize HIF-1α even under normoxic conditions (Isaacs et al., 2005; Selak et al., 2005). These oncometabolites illustrate how mitochondrial metabolic dysfunction can directly influence nuclear transcriptional programs.

Gastric cancer exhibits substantial heterogeneity in histopathological features, molecular characteristics, and clinical behavior, necessitating sophisticated classification systems to guide treatment decisions (Cristescu et al., 2015; Tan and Yeoh, 2015). The Lauren classification, established in 1965, divides gastric cancer into intestinal and diffuse types based on histological features, with the intestinal type characterized by cohesive gland-forming cells and the diffuse type showing signet ring cells with dispersed growth patterns (Lauren, 1965). This classification retains prognostic relevance, as diffuse-type gastric cancer is associated with younger age, advanced stage at diagnosis, and poorer outcomes.

The Asian Cancer Research Group (ACRG) proposed an alternative molecular classification based on gene expression profiling, dividing gastric cancer into microsatellite stable/epithelial-mesenchymal transition (MSS/EMT), MSS/p53+, MSS/p53-, and MSI subtypes (Cristescu et al., 2015). This classification system demonstrated prognostic significance, with MSS/EMT tumors showing the worst outcomes and MSI tumors the best survival. Integration of these molecular classifications with clinical and pathological features enables more precise patient stratification and identification of therapeutic vulnerabilities.

*Helicobacter pylori* infection represents the primary risk factor for gastric cancer, with approximately 89% of non-cardia gastric cancers attributable to this bacterial infection. The bacterium promotes carcinogenesis through multiple mechanisms including chronic inflammation, direct genotoxic effects of bacterial virulence factors (CagA and VacA), and alteration of gastric epithelial cell signaling (Cover and Blanke, 2005; Polk and Peek, 2010). VacA, a pore-forming toxin, directly targets mitochondria, inducing cytochrome c release, mitochondrial fragmentation, and activation of apoptosis and autophagy pathways. These mitochondrial effects contribute to the gastric pathology induced by *H. pylori* infection.

Mitochondrial dysfunction has emerged as a hallmark of cancer, contributing to the metabolic reprogramming, resistance to apoptosis, and altered redox homeostasis characteristic of malignant cells (Hanahan and Weinberg, 2011; Pavlova and Thompson, 2016). Unlike the original Warburg hypothesis suggesting that cancer arises from defective respiration, contemporary understanding recognizes that mitochondrial dysfunction in cancer is complex and context-dependent, with some cancers showing impaired OXPHOS while others maintain or even enhance oxidative metabolism (Bao et al., 2021).

mtDNA mutations represent a major mechanism of mitochondrial dysfunction in cancer. These mutations can be classified as homoplasmic (all mtDNA copies identical) or heteroplasmic (mixture of wild-type and mutant mtDNA), with the mutation load determining functional consequences. Mutations in protein-coding genes typically impair electron transport chain function, while mutations in tRNA genes can affect mitochondrial protein synthesis more broadly. The threshold effect describes the phenomenon wherein mitochondrial dysfunction becomes apparent only when mutant mtDNA exceeds a critical level, typically 60–90% of total mtDNA (Rossignol et al., 2003; Stewart and Chinnery, 2015).

Germline mtDNA mutations may cause a diverse array of mitochondrial diseases, many of which increase cancer risk. For example, mutations in succinate dehydrogenase subunits cause hereditary paraganglioma-pheochromocytoma syndrome, demonstrating the direct link between mitochondrial dysfunction and tumorigenesis (Baysal et al., 2000; Neumann et al., 2002; Kim et al., 2022). Somatic mtDNA mutations are even more common in sporadic cancers, with studies documenting mtDNA mutations in approximately 50–80% of solid tumors.

Beyond mtDNA mutations, alterations in nuclear-encoded mitochondrial proteins contribute to cancer development. Mutations in succinate dehydrogenase (SDH) and fumarate hydratase (FH) cause accumulation of succinate and fumarate, respectively, which inhibit α-ketoglutarate-dependent dioxygenases including prolyl hydroxylases that regulate HIF-1α stability (Pollard et al., 2005; King et al., 2006). This pseudohypoxic signaling promotes angiogenesis and metabolic reprogramming independent of oxygen levels. Similarly, mutations in isocitrate dehydrogenase 1 and 2 (IDH1/2) produce the oncometabolite 2-hydroxyglutarate, which alters DNA and histone methylation patterns (Dang et al., 2009; Ward et al., 2013).

Mitochondrial dynamics are frequently altered in cancer, with changes in fission-fusion balance affecting metabolic plasticity, cell migration, and therapeutic resistance. Increased mitochondrial fission, mediated by DRP1 activation, promotes glycolytic metabolism and is associated with cancer cell invasion and metastasis. Conversely, mitochondrial fusion supports OXPHOS and is associated with cancer stem cell maintenance (Zhao et al., 2011; Xie et al., 2015; Tan et al., 2025).

## 3 Mitochondrial DNA mutations in gastric cancer

Somatic mutations in mitochondrial DNA occur frequently in gastric cancer, with mutation frequencies ranging from 46% to 82% across different studies. These mutations affect both coding and non-coding regions of the mitochondrial genome, with distinct mutation patterns compared to nuclear DNA mutations. The most common mtDNA mutations in gastric cancer include point mutations, deletions, and insertions, with the D-loop regulatory region showing particularly high mutation frequency (Burgart et al., 1995; Chang et al., 2023).

To accurately evaluate the biological role of these alterations, it is critical to differentiate true driver mutations from passenger mutations within the mitochondrial genome. Passenger mtDNA mutations are thought to accumulate during tumorigenesis as a consequence of oxidative stress, rapid clonal expansion, or genetic drift. They are often neutral low-heteroplasmy variants and may have little or no measurable effect on OXPHOS efficiency or cellular phenotype (Guo et al., 2013). In contrast, driver mtDNA mutations are typically recurrent and often affect conserved residues in electron transport chain components, particularly Complex I subunits such as ND1, ND4, and ND5, or occur in regulatory regions. Such alterations generally reach high heteroplasmy levels, and in some cases homoplasmy, consistent with positive selection during tumor evolution (Lin et al., 2024). These driver mutations suggest disrupt respiratory chain complex assembly or catalytic function, elevate ROS generation, and trigger retrograde nuclear reprogramming to promote tumor cell survival and metabolic adaptation. However, as noted in recent functional characterizations, identifying true driver mtDNA variants requires rigorous functional validation, such as cybrid modeling, rather than observational sequencing alone. This is because even high-load mutations can have context-dependent effects, and in some tumors, they may even appear tumor-suppressive depending on metabolic state and bioenergetic demand (Miller et al., 2025).

Heteroplasmy the coexistence of mutant and wild-type mtDNA is a key determinant of mitochondrial genetic behavior in gastric cancer. The phenotypic impact of an mtDNA mutation depends strongly on its heteroplasmy level, often following a threshold effect in which OXPHOS impairment, increased ROS production, and retrograde signaling become apparent only when mutant load exceeds a critical level; however, this threshold is mutation- and tissue-dependent rather than universal (Smith et al., 2022). Low-heteroplasmy mtDNA variants are often functionally buffered and may behave as neutral passenger mutations, whereas high-load or homoplasmic mutations are more likely to drive metabolic reprogramming. In gastric cancer, heteroplasmy may also shape clinical behavior by influencing redox balance, therapeutic response, and tumor aggressiveness, although the strength of these associations varies across cohorts and individual variants (Picard et al., 2014).

Recent whole mitochondrial genome sequencing studies in gastric cancer have expanded our understanding of clinically relevant mtDNA alterations, revealing recurrent variants in Complex I–related genes, particularly MT-ND1, MT-ND4, and MT-ND5, as well as in the D-loop and tRNA regions. Some cohorts further suggest that mtDNA mutation burden and heteroplasmy patterns may vary across clinicopathologic subgroups, although these associations remain less consistent and require validation in larger studies (Cavalcante et al., 2019). Several patient-derived mtDNA alterations have been functionally interrogated in model systems such as cybrids and mtDNA-depleted ρ0\rho 0ρ0 cells, supporting their direct effects on OXPHOS efficiency, ROS generation, and related stress-response pathways. In gastric cancer specifically, however, most primary evidence still comes from sequencing and clinicopathologic association studies, so the extent of functional validation remains limited for individual variants (Lee et al., 2014). Together, these patient-based genomic and functional studies underscore the mechanistic contribution of mtDNA mutations to gastric cancer progression.

The resulting mitochondrial dysfunction triggers retrograde signaling that reprograms nuclear gene expression. Studies using cytoplasmic hybrid (cybrid) cells, created by fusing mitochondria from gastric cancer cells with ρ0 cells (lacking mtDNA), demonstrate that mtDNA mutations alone can alter nuclear gene expression patterns and promote tumorigenic phenotypes (Cruz-Bermúdez et al., 2015). These cybrid studies provide compelling evidence for the causal role of mtDNA mutations in cancer development through retrograde signaling mechanisms.

mtDNA mutations initiate retrograde signaling cascades through multiple mechanisms. Reduced electron transport chain efficiency leads to membrane depolarization, which activates calcium signaling and other stress responses. Increased ROS production directly activates transcription factors including NF-κB, HIF-1α, and NRF2, initiating adaptive transcriptional programs. Metabolic alterations, including changes in NAD+/NADH ratio and accumulation of TCA cycle intermediates, further modulate nuclear transcription through effects on chromatin-modifying enzymes (Katada et al., 2012).

The retrograde response to mtDNA mutations includes upregulation of glycolytic enzymes, creating the Warburg phenotype even in the absence of hypoxia. HIF-1α stabilization by mitochondrial ROS or accumulated succinate activates transcription of glycolytic genes including glucose transporters (*GLUT1*, *GLUT3*), hexokinases (*HK1*, *HK2*), and lactate dehydrogenase A (*LDHA*) (Kim et al., 2006). This metabolic reprogramming enables cancer cells to maintain ATP production despite impaired OXPHOS while providing biosynthetic intermediates for rapid proliferation.

mtDNA mutations also promote resistance to apoptosis, a hallmark of cancer that contributes to therapeutic resistance. Mitochondrial dysfunction can impair cytochrome c release in response to apoptotic stimuli, while alterations in mitochondrial dynamics affect sensitivity to cell death signals (Kroemer and Pouyssegur, 2008; Galluzzi et al., 2012). The reduced apoptotic potential of cells with mtDNA mutations provides a survival advantage during malignant transformation and contributes to the resistance of gastric cancers to chemotherapy and radiation.

Although numerous studies support a tumor-promoting role for mtDNA mutations in gastric cancer, several reports highlight conflicting or context-dependent findings. Some mtDNA mutations particularly those present at low heteroplasmy levels behave as neutral passenger variants without measurable effects on OXPHOS activity or ROS generation (Hahn and Zuryn, 2019). In addition, certain mutations in Complex I or mitochondrial tRNA genes have been shown to reduce mitochondrial ROS output, thereby attenuating retrograde signaling and failing to enhance malignant phenotypes in cybrid models (Hahn and Zuryn, 2019). Population-based genomic and Mendelian randomization studies have also demonstrated that certain mtDNA alterations such as changes in mtDNA copy number do not correlate with gastric cancer risk, tumor stage, or clinical progression, suggesting that not all mitochondrial genomic changes contribute directly to carcinogenesis (Zhou et al., 2025).

Notably, the functional impact of mtDNA mutations on gastric carcinogenesis is not entirely unidirectional, and several studies report paradoxical inhibitory effects on tumor progression. While heteroplasmic or mild missense mtDNA mutations generally promote tumorigenesis by enhancing ROS generation and activating oncogenic retrograde signaling, severe homoplasmic defects or truncating mutations can lead to profound OXPHOS collapse. This phenomenon has been demonstrated in cybrid models harboring pathogenic tRNALys mutations such as G8363A, which markedly impair respiratory chain enzyme activities, oxygen consumption, and mitochondrial protein synthesis, resulting in severe disruption of oxidative phosphorylation (Bornstein et al., 2005). Such severe mitochondrial impairment may constrain tumor growth and metastatic potential, because proliferating cancer cells still require a minimal level of mitochondrial function to sustain essential biosynthetic processes, including *de novo* pyrimidine synthesis via DHODH and the generation of TCA cycle–derived intermediates (Zhou et al., 2021). Furthermore, certain high-load mtDNA mutations have been reported to sensitize gastric cancer cells to oxidative stress–induced apoptosis and, in some settings, to chemotherapy (Lee et al., 2014). Collectively, these findings indicate that the biological consequences of mtDNA mutations depend heavily on mutation type, heteroplasmy threshold, metabolic context, and tumor stage, underscoring the need for functional assays rather than sequence-based interpretation alone when evaluating their oncogenic relevance. To provide a concise overview of the most frequently reported mitochondrial alterations in gastric cancer, we summarized the recurrent mtDNA mutations and their clinical relevance in **Table 1**.

**Table 1:** Recurrent mtDNA alterations reported in gastric cancer and their clinical relevance.

Gene/region	Common alteration	Functional impact	Clinical significance	Ref
D-loop (non-coding)	High-frequency point mutations; instability in hypervariable region (including D310)	Altered mtDNA replication/transcription; increased ROS	Frequently observed in gastric cancer; may relate to tumor progression and early detection	(Wu et al., 2005)
D-loop (non-coding)	~50-bp deletion within replication origin region	Reduced mtDNA replication/transcription; elevated ROS	Reported in gastric cancer and associated in some cohorts with advanced disease	(Burgart et al., 1995; Lee et al., 2014)
Complex I genes (ND1, ND4, ND5)	Recurrent mutations in mtDNA-encoded Complex I subunits	Impaired Complex I function; increased ROS; shift toward glycolysis	Linked to mitochondrial dysfunction in gastric cancer; clinical impact is suggested but not always consistent across cohorts	(Wu et al., 2005)
MT-RNR1/tRNA regions	Heteroplasmic substitutions and recurrent variants	May impair mitochondrial translation and respiratory efficiency	Reported in gastric cancer whole-mtGenome studies	(Cavalcante et al., 2019)
mtDNA copy number	Decrease or depletion	Mitochondrial dysfunction and altered bioenergetics	Decreased mtDNA copy number observed in gastric cancer, especially in advanced stages in some cohorts	(Wen et al., 2013)

### 3.1 Mitochondrial signaling and the tumor microenvironment in gastric cancer

Mitochondrial dysfunction in gastric cancer cells profoundly reshapes the tumor microenvironment (TME) by altering metabolic fluxes, redox homeostasis, and intercellular signaling networks (Liu et al., 2026). Elevated mitochondrial ROS and retrograde signaling not only reprogram cancer cell metabolism but also exert paracrine effects on surrounding stromal and immune cells, establishing a microenvironment that promotes tumor progression and immune evasion (Kuo et al., 2022).

Immune cell function is tightly coupled to mitochondrial metabolism, and mitochondrial signals originating from cancer cells directly influence immune cell fate. High ROS levels and lactate accumulation within the TME impair mitochondrial respiration in cytotoxic T cells and NK cells, driving metabolic exhaustion and reducing antitumor activity (Tu et al., 2021). In contrast, regulatory T cells (Tregs) and tumor-associated macrophages (TAMs) adapt to these hostile conditions by shifting toward OXPHOS-dependent or fatty-acid–driven metabolic programs, enabling them to maintain suppressive phenotypes in hypoxic, nutrient-restricted environments (Pacella et al., 2018; Su et al., 2020).

Cancer cells can induce oxidative stress and mitochondrial dysfunction in adjacent cancer-associated fibroblasts (CAFs), leading to a glycolytic, catabolic stromal phenotype characterized by increased lactate and pyruvate production (Guido et al., 2012). These CAF-derived metabolites can be transferred to cancer cells and support mitochondrial metabolism and tumor growth. Hypoxia further amplifies this metabolic crosstalk by stabilizing HIF-1α, reducing mitochondrial oxidative metabolism, and promoting glycolytic reprogramming in both tumor and stromal compartments (Koido et al., 2017). In parallel, mitochondrial dysfunction and ROS-dependent signaling can activate inflammatory pathways that contribute to an immunosuppressive microenvironment and tumor progression in gastric cancer (Avagliano et al., 2018).

### 3.2 ROS-mediated activation of NF-κB and HIF-1α

Reactive oxygen species generated at complexes I and III of the electron transport chain act as key signaling molecules under physiological conditions, whereas excessive ROS contribute to oxidative damage and cellular dysfunction (Brand, 2010; Bleier and Dröse, 2013). Superoxide (O_2_•^-^) produced by electron leakage is rapidly converted to hydrogen peroxide (H_2_O_2_) by MnSOD (SOD2) in the matrix or Cu/ZnSOD (SOD1) in the intermembrane space. H_2_O_2_, being stable and membrane-permeable, serves as the principal mediator of mitochondrial redox signaling, oxidizing cysteine residues in target proteins and transmitting oxidative cues to the cytosol and nucleus (Dickinson and Chang, 2011; Forman et al., 2014). mtDNA mutations that impair electron transport chain activity further elevate ROS levels by increasing reduced electron carriers, destabilizing complex assembly, and expanding mitochondrial mass as a compensatory response.

NF-κB is a family of transcription factors regulating inflammation, immunity, cell survival, and proliferation. In the canonical pathway, the p50/p65 heterodimer is retained in the cytoplasm by IκB proteins until IKK-mediated phosphorylation triggers IκB degradation and NF-κB nuclear translocation. Mitochondrial ROS activate NF-κB both directly through oxidation of IKK and related components and indirectly via upstream kinases such as PKC, PI3K, and MAPKs (Schulze‐Osthoff et al., 1998). In gastric cancer, H. pylori infection amplifies ROS-driven NF-κB activation: CagA enhances ROS-generating pathways, while VacA disrupts mitochondrial membrane integrity. Sustained NF-κB signaling induces pro-inflammatory cytokines (IL-6, IL-8, TNF-α) and anti-apoptotic genes (BCL-2, BCL-XL, cIAP1/2, XIAP), promoting proliferation, invasion, and resistance to cell death (Kucharczak et al., 2003; Basseres and Baldwin, 2006).

**Figure 1:**
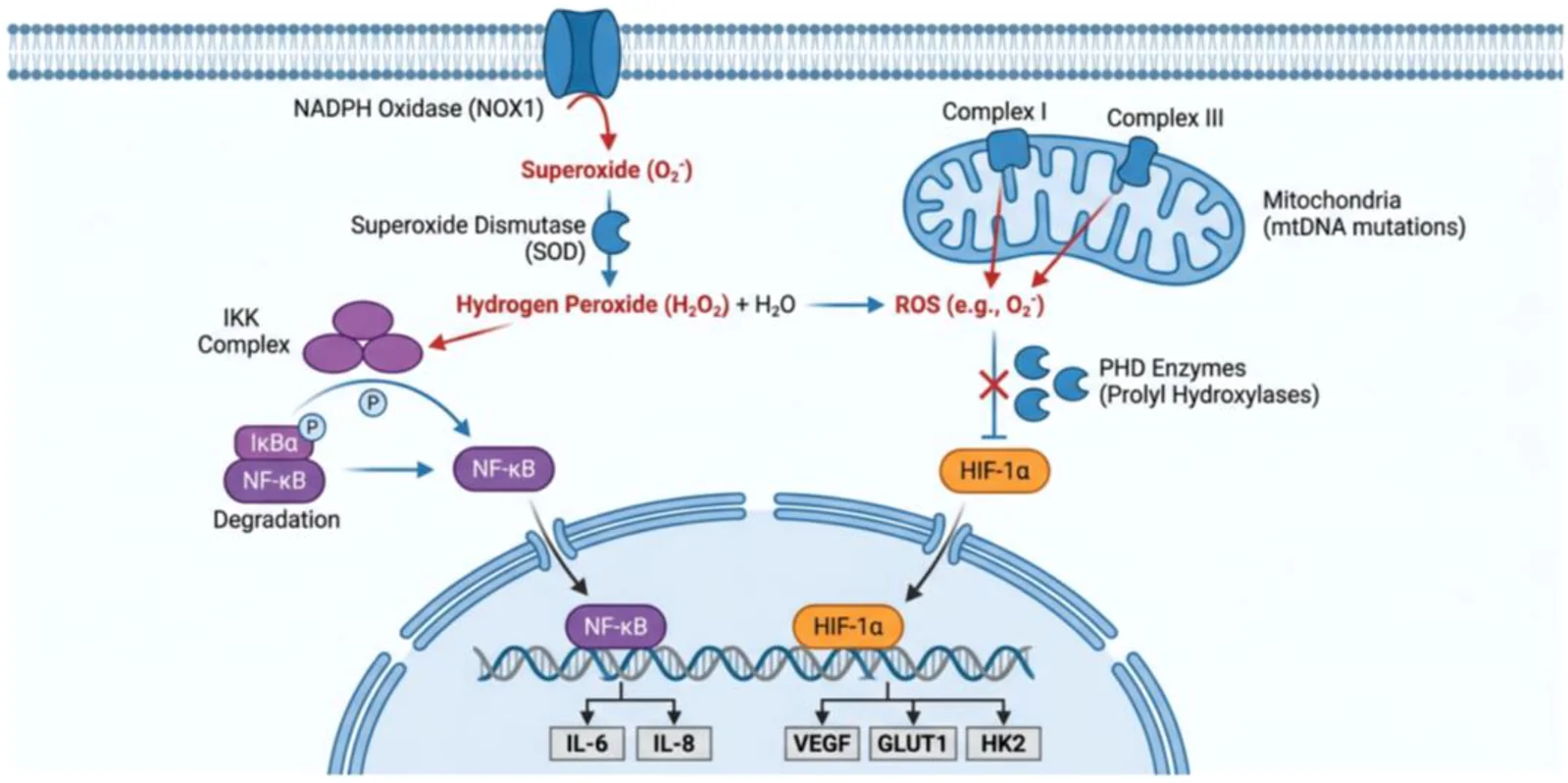
ROS-mediated activation of NF-κB and HIF-1α signaling pathways. This schematic summarizes how reactive oxygen species (ROS) promote transcriptional reprogramming in gastric cancer. (1) NOX1 at the plasma membrane generates superoxide (O_2_•^-^). (2) Mitochondrial complexes I and III produce ROS, especially when mtDNA mutations impair electron transport. (3) Superoxide is converted to hydrogen peroxide (H_2_O_2_) by SOD enzymes. (4) H_2_O_2_ activates the IKK complex, triggering IκBα degradation and NF-κB (p50/p65) nuclear translocation. (5) ROS inhibit PHD enzymes, preventing HIF-1α hydroxylation and degradation, resulting in HIF-1α stabilization. (6) NF-κB and HIF-1α induce transcription of pro-inflammatory cytokines (IL-6, IL-8), angiogenic factors (VEGF), and glycolytic enzymes (GLUT1, HK2, LDHA).

Mitochondrial retrograde signaling profoundly reshapes nuclear transcriptional landscapes. Glycolytic enzymes (HK2, PFK, PKM2, LDHA), glucose transporters (GLUT1, GLUT3), and pentose phosphate pathway enzymes are upregulated, shifting metabolism toward aerobic glycolysis and enhancing biosynthetic capacity. Anti-apoptotic genes (BCL-2 family, IAPs, FLIP) confer resistance to cell death and therapeutic stress (Kucharczak et al., 2003). NF-κB-driven cytokines (IL-6, IL-8, TNF-α) establish a chronic inflammatory microenvironment that synergizes with H. pylori-induced oxidative stress to promote gastric carcinogenesis (Greten et al., 2004; Karin and Greten, 2005). (**Figure 1**).

## 4 Warburg phenotype amplification

The Warburg effect, first described by Otto Warburg in 1924, refers to the preference of cancer cells for aerobic glycolysis despite the higher efficiency of oxidative phosphorylation (OXPHOS). Although most cancer cells retain functional mitochondria, enhanced glycolysis remains a consistent metabolic feature across many tumors (Koppenol et al., 2011). Rather than indicating irreversible mitochondrial damage, the Warburg effect reflects an adaptive reprogramming that supports rapid ATP generation, supplies biosynthetic precursors, and shapes a microenvironment favorable for tumor progression.

Cancer metabolism is highly flexible, with cells shifting between glycolysis and OXPHOS depending on nutrient availability, oxygen tension, and genetic background (Faubert et al., 2020). Cells harboring mtDNA mutations or residing in hypoxic niches rely more heavily on glycolysis, whereas others maintain or increase OXPHOS using substrates such as glutamine and fatty acids. This metabolic plasticity enables survival under diverse microenvironmental stresses.

Mitochondrial retrograde signaling contributes directly to Warburg phenotype amplification in gastric cancer. ROS generated by dysfunctional mitochondria stabilize HIF-1α, which upregulates glycolytic enzymes and glucose transporters, promoting aerobic glycolysis even under normoxia. HIF-1α also induces pyruvate dehydrogenase kinase 1 (PDK1), which inhibits the pyruvate dehydrogenase complex and blocks pyruvate entry into mitochondria, reinforcing glycolytic flux (Papandreou et al., 2006; McFate et al., 2008). Upregulation of LDHA further drives conversion of pyruvate to lactate, regenerating NAD+ and sustaining glycolysis. Lactate export via MCT1/4 acidifies the tumor microenvironment, promoting invasion, immune evasion, and therapeutic resistance.

Beyond HIF-1α, transcription factors such as c-Myc synergize with retrograde signaling to enhance glycolysis and glutamine metabolism, supporting mitochondrial anaplerosis and biosynthesis. Glycolytic intermediates feed essential anabolic pathways, including the pentose phosphate pathway for nucleotide synthesis and NADPH production, amino acid biosynthesis, and lipid formation (Wise et al., 2008; Patra and Hay, 2014).

The acidic microenvironment generated by lactate export promotes extracellular matrix degradation, suppresses cytotoxic immune responses, and contributes to chemoradiotherapy resistance (Correia and Bissell, 2012; Estrella et al., 2013). These metabolic and microenvironmental changes collectively support tumor growth and progression.

**Figure 2:**
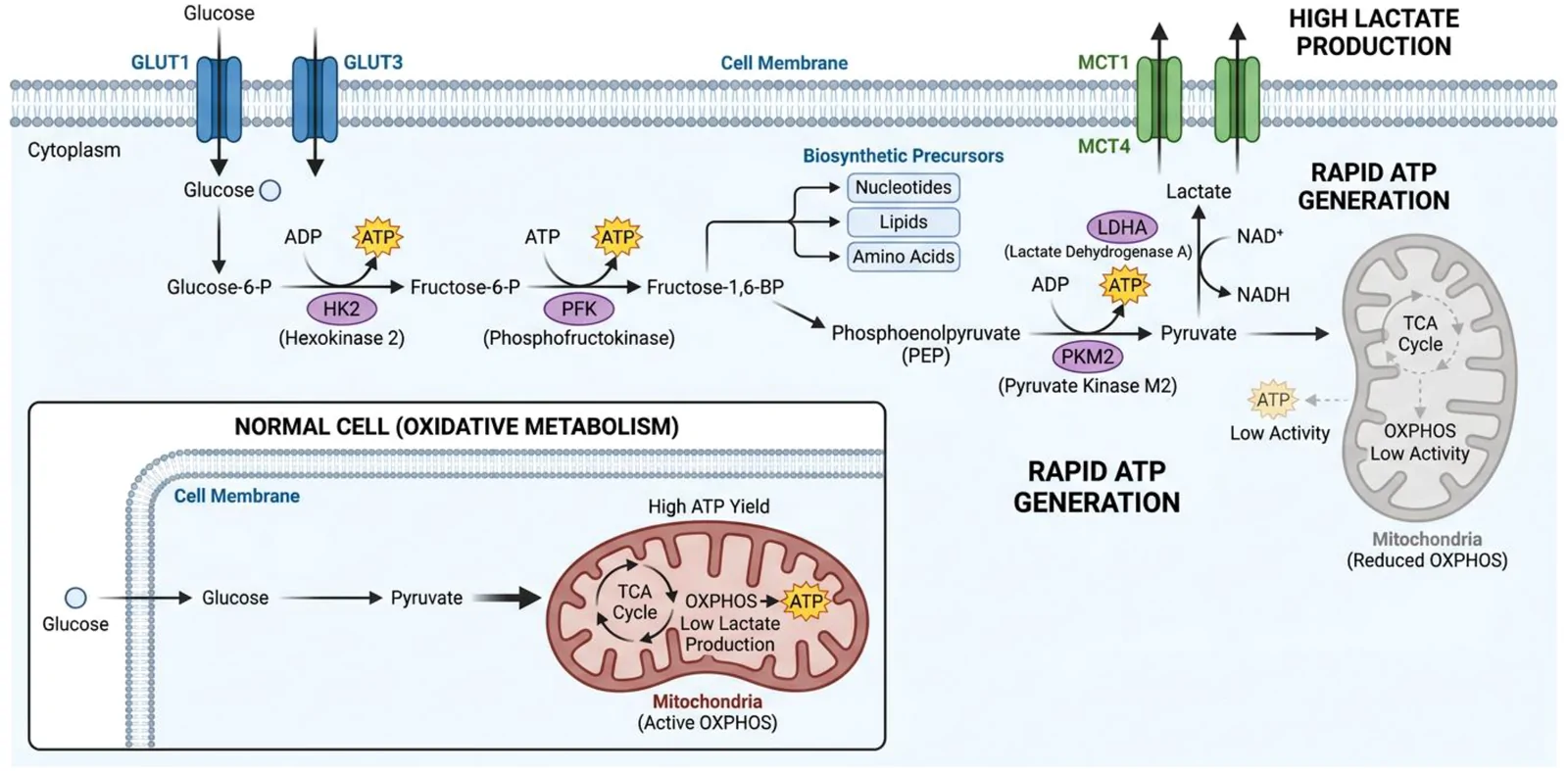
The Warburg effect in gastric cancer cells. Gastric cancer cells exhibit increased glucose uptake through GLUT1/3 and enhanced glycolysis driven by HK2, PFK, PKM2, and LDHA, with lactate export via MCT1/4. In contrast to normal cells where pyruvate enters mitochondria for OXPHOS, gastric cancer cells suppress mitochondrial respiration and divert glycolytic intermediates toward biosynthetic pathways that support rapid proliferation.

Therapeutically, targeting glycolysis or lactate metabolism offers promising strategies for gastric cancers exhibiting the Warburg phenotype. Inhibitors of HK2, LDHA, or MCT transporters can disrupt glycolytic flux and reduce acidosis (Le et al., 2010; Stine et al., 2015). Metformin, an inhibitor of complex I, further increases energetic stress in cancer cells with mitochondrial dysfunction and activates AMPK to suppress mTOR signaling, demonstrating selective vulnerability in tumors dependent on retrograde signaling pathways (Kasznicki et al., 2014; Cazzaniga and Bonanni, 2015) (**Figure 2**).

## 5 Mitochondrial DNA release, cGAS–STING activation, and extracellular vesicle-mediated communication

Mitochondrial dysfunction not only generates ROS-dependent retrograde signals but also promotes the release of mtDNA, which serves as a potent activator of innate immune pathways. Under conditions of mitochondrial stress, impaired mitophagy, or outer membrane permeabilization, mtDNA can leak into the cytosol through mechanisms involving VDAC oligomerization, BAX/BAK pore formation, or incomplete clearance of damaged mitochondria. Cytosolic mtDNA is recognized by the cyclic GMP–AMP synthase (cGAS), which catalyzes the formation of cGAMP and activates the adaptor protein STING at the endoplasmic reticulum (Kim et al., 2023; Liu et al., 2025). Activated STING triggers TBK1-mediated phosphorylation of IRF3, leading to type I interferon production and pro-inflammatory gene expression. This mtDNA-dependent cGAS–STING signaling represents a second major retrograde pathway that links mitochondrial damage to nuclear transcriptional reprogramming in gastric cancer (Tanaka and Chen, 2012; Tian et al., 2024).

In addition to cytosolic leakage, mtDNA and other mitochondrial components can be exported extracellularly through extracellular vesicles (EVs), including exosomes and microvesicles. Cancer cells experiencing mitochondrial stress release EVs containing mtDNA fragments, mitochondrial proteins, and even small mitochondrial structures (Scheid et al., 2021). These vesicles can be taken up by neighboring cancer cells, stromal fibroblasts, or immune cells, thereby propagating mitochondrial stress signals across the tumor microenvironment. EV-mediated mitochondrial communication has been shown to modulate inflammatory responses, enhance metabolic adaptation, and promote immune evasion, adding an additional layer of complexity to mitochondrial retrograde signaling (Di Mambro et al., 2023).

Together, mtDNA release, cGAS–STING activation, and EV-mediated mitochondrial communication complement ROS-dependent pathways and form an integrated network through which dysfunctional mitochondria influence nuclear gene expression, inflammation, and tumor progression in gastric cancer.

## 6 Therapeutic targeting of mitochondrial retrograde signaling

The integrated model of mitochondrial retrograde signaling highlights several actionable therapeutic nodes in gastric cancer. Mitochondria-targeted antioxidants such as MitoQ and SkQ1 can attenuate mitochondrial ROS production, thereby suppressing NF-κB activation, reducing HIF-1α stabilization, and interrupting the positive feedback loop that drives glycolytic reprogramming (Wu and Wu, 2026). Pharmacologic inhibition of OXPHOS particularly Complex I inhibitors such as IACS-010759 or phenformin can exploit metabolic vulnerabilities created by pathogenic mtDNA mutations or high-heteroplasmy defects that shift tumor cells toward glycolysis (Skemiene et al., 2020; Yap et al., 2023).

Targeting downstream transcriptional mediators of retrograde signaling offers additional therapeutic opportunities. NF-κB inhibitors (including IKK inhibitors) may reduce inflammatory signaling and apoptosis resistance, while HIF-1α inhibitors such as PX-478 or acriflavine can suppress glycolytic gene expression and reverse the Warburg phenotype (Sokolova and Naumann, 2017; Zhao et al., 2024). These agents may be particularly effective in tumors where mtDNA mutations drive ROS-mediated stabilization of HIF-1α and NF-κB.

Emerging precision oncology strategies aim to selectively target mitochondrial metabolic dependencies. Synthetic-lethality approaches that exploit OXPHOS impairment, metabolic inhibitors that target glycolysis or lactate metabolism, and combination regimens pairing OXPHOS inhibitors with chemotherapy have shown promise in preclinical models. Biomarkers such as mtDNA mutation burden, heteroplasmy levels, circulating mtDNA, and activation markers of NF-κB or HIF-1α may help identify patients most likely to benefit from mitochondria-targeted therapies (Doherty and Cleveland, 2013; Pérez-Amado et al., 2021). Metformin has also demonstrated direct anti-tumor activity in gastric cancer, including inhibition of EMT markers (vimentin, β-catenin) and induction of E-cadherin, along with reduced migration and invasion of AGS cells under both normoglycemic and hyperglycemic conditions (Chen et al., 2015). Additional studies further support its anti-proliferative and metabolic effects in gastric cancer models (Valaee et al., 2017). Although these therapeutic strategies are promising, several limitations constrain their clinical translation. Most mitochondria-targeted agents remain in preclinical or early clinical stages, and both HIF-1α and NF-κB inhibitors face narrow therapeutic windows and toxicity concerns. Glycolysis inhibitors also show dose-limiting adverse effects, and clinical evidence for metformin in gastric cancer remains mixed. Combination approaches such as pairing OXPHOS inhibitors with chemotherapy are conceptually attractive but still exploratory. These considerations highlight that mitochondria-focused therapies are at an early developmental stage and require further validation before routine clinical use.

To provide a concise overview of the major therapeutic nodes within mitochondrial retrograde signaling and their representative agents, we summarized key interventions in **Table 2**, highlighting their mechanisms of action and expected biological effects in gastric cancer.

**Table 2:** Therapeutic strategies targeting mitochondrial retrograde signaling and metabolic adaptations in gastric cancer.

Target/node	Representative agents	Main mechanism	Expected biological effect	Ref
Mitochondrial ROS	MitoQ, SkQ1	Scavenging of mitochondrial ROS	Suppresses ROS-driven and may blocks Warburg	(Rondeau et al., 2024)
Complex I/OXPHOS	IACS-010759, phenformin, metformin	Complex I inhibition/OXPHOS suppression	Exploits OXPHOS dependence, lowers ATP production, impairs tumor growth in OXPHOS-addicted cancers	(Yap et al., 2023)
HIF-1α pathway	PX-478, acriflavine, digoxin	Inhibition of HIF-1α translation/dimerization/function	Downregulates HIF-1 target genes and glycolytic reprogramming; can suppress Warburg-like adaptation	(Welsh et al., 2004)
Lactate & MCTs	AZD3965, MCT1 inhibitors	Blockade of lactate transport	Disrupts lactate shuttling and tumor–stroma metabolic coupling	(Bola et al., 2014)

## 7 Progress and emerging directions in mitochondrial retrograde signaling

Mitochondrial dysfunction has increasingly been recognized as a central driver of gastric carcinogenesis, with somatic mtDNA mutations contributing to impaired electron transport chain activity and excessive ROS generation. These mitochondrial stress signals activate retrograde pathways that converge on key transcriptional regulators, most notably NF-κB and HIF-1α, which orchestrate pro-inflammatory, pro-survival, and metabolic reprogramming programs (Ghosh et al., 1998; Rius et al., 2008). The sustained activation of NF-κB by mitochondrial ROS promotes chronic inflammation and cellular survival, particularly in the context of Helicobacter pylori infection, which synergistically enhances NF-κB signaling and fosters a tumor-promoting microenvironment. In parallel, ROS-mediated stabilization of HIF-1α under normoxic conditions enables cancer cells to adopt hypoxia-like transcriptional profiles, driving glycolytic metabolism, angiogenesis, and metastatic potential (Chandel et al., 2000; Jing et al., 2019).

These retrograde signaling pathways collectively reinforce the Warburg phenotype, shifting cellular metabolism toward aerobic glycolysis and lactate production despite adequate oxygen availability (Vander Heiden et al., 2009; Liberti and Locasale, 2016; Jiang and Ye, 2025). This metabolic reprogramming not only supports rapid proliferation through enhanced biosynthetic flux but also contributes to acidification of the tumor microenvironment, facilitating invasion and immune evasion. Importantly, emerging evidence indicates that mitochondrial retrograde signaling is not merely a downstream consequence of metabolic stress but functions as an active, causal mechanism that shapes the transcriptional and metabolic landscape of gastric cancer cells.

## 8 Conclusion

Mitochondrial retrograde signaling represents a fundamental mechanism linking mitochondrial dysfunction to gastric carcinogenesis. Somatic mtDNA mutations, occurring in approximately half of gastric cancers, disrupt electron transport chain function and increase ROS production, initiating signaling cascades that activate NF-κB and stabilize HIF-1α. These transcription factors reprogram nuclear gene expression to promote inflammation, metabolic reprogramming toward aerobic glycolysis, and resistance to apoptosis, creating a pro-tumorigenic cellular state. The Warburg phenotype in gastric cancer is actively amplified by mitochondrial retrograde signaling, providing metabolic advantages for cancer cell proliferation and survival. Rather than being a passive consequence of defective respiration, the shift toward aerobic glycolysis is driven by HIF-1α activation in response to mitochondrial ROS, even in oxygen-replete conditions, supporting rapid proliferation and fostering an acidic microenvironment that promotes invasion and immune evasion.

Understanding these mechanisms identifies multiple potential therapeutic targets. Inhibition of NF-κB or HIF-1α could interrupt retrograde signaling and reverse oncogenic gene expression programs, while targeting glycolytic enzymes or lactate metabolism may exploit the metabolic dependence of gastric cancers with mitochondrial dysfunction. Mitochondria-targeted antioxidants could reduce ROS production and interrupt signaling cascades at their source. Integrating mitochondrial biology with cancer genetics and metabolism provides a comprehensive framework for understanding gastric carcinogenesis. As therapeutic strategies targeting these pathways enter clinical development, biomarker-driven patient selection will be essential. Future research characterizing the heterogeneity of mitochondrial function within tumors and developing improved preclinical models will accelerate translation of mechanistic insights into improved outcomes for patients with gastric cancer.
